# Understanding and managing sight-threatening diabetic retinopathy

**Published:** 2023-07-07

**Authors:** David Yorston

**Affiliations:** 1 Consultant Ophthalmologist, Tennent Institute of Ophthalmology, Gartnavel Hospital, Glasgow Scotland.

**Diabetic retinopathy can cause blindness. However, with prompt recognition and treatment, eye workers can prevent severe sight loss in most patients.**

**Figure:**
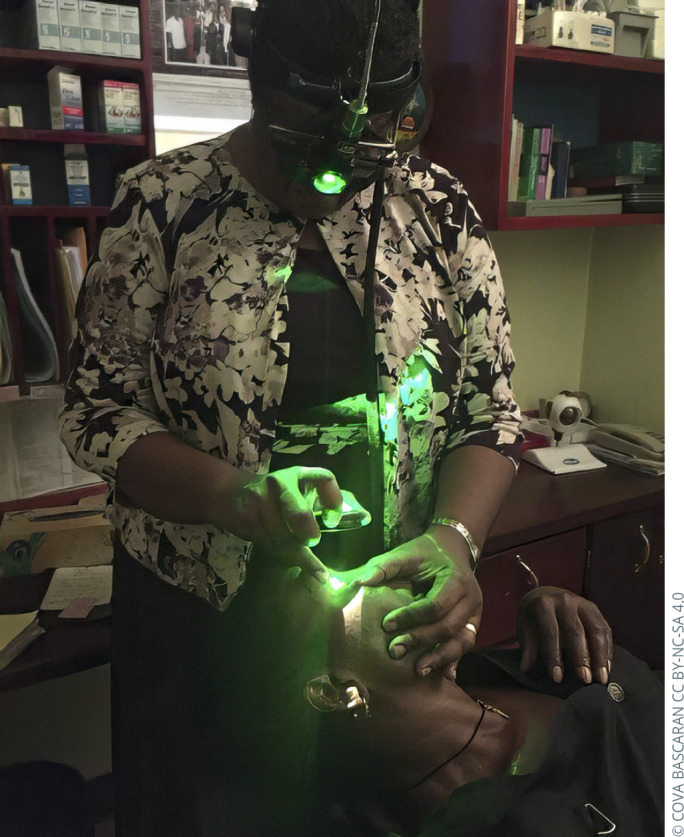
Indirect laser delivery for diabetic retinopathy. **DOMINICA**

Ideally, we would prevent diabetic retinopathy (DR) using either primary prevention, by reducing the risk of Type 2 diabetes, or secondary prevention: managing diabetes so effectively that the risk of complications is reduced. Eye workers should use every opportunity to emphasise the importance of maintaining a healthy weight to prevent Type 2 diabetes, as well as achieving good control of blood sugar and blood pressure to reduce the risk of developing severe retinopathy. However, given that there are many millions of people with diabetes, we will have to manage established DR to minimise the risk of losing vision.

Early recognition and diagnosis of DR has a major impact on the effectiveness of treatment. Because the management of DR is mostly about preserving vision, rather than restoring sight, this means that the better the vision is when treatment is started, the better it will be when treatment is complete.

## How does diabetic retinopathy develop?

Diabetes leads to loss of the pericytes – the cells that support the retinal capillaries. As these cells are lost, the capillaries start to leak. Normally, the cells of the retinal capillaries are connected by “tight junctions”. This means that they are impermeable, except to small molecules like water, glucose, oxygen, and carbon dioxide. The effect of this is to suck water out of the retina, so that, in a normal eye, the retina contains very little water outside of the retinal cells.

In patients with diabetes, the permeable capillaries allow larger molecules (e.g. proteins and lipids) to leak into the retina, resulting in hard exudates. These large molecules draw water out of the capillaries and the retina becomes swollen and waterlogged, or oedematous. If the retinal oedema involves the macula, it is known as **macular oedema** (see below). If this does not involve the fovea – the central portion of the macula – it will not affect the vision. However, if the fovea is involved, visual acuity will be reduced.

As the damage to the capillaries continues, they become blocked. This leads to inadequate blood supply (ischaemia). The ischaemic retina produces local growth factors to encourage new blood vessels to grow to compensate for the blocked capillaries. This is known as **proliferative diabetic retinopathy** (see below). Unfortunately, these new vessels do not grow in the retina, but on the back of the vitreous face. This leads to sheets of fibrovascular tissue attached to both the retina and the vitreous. As this fibrous tissue contracts, it tears the blood vessels, causing vitreous haemorrhage, and pulls on the retina, causing a traction retinal detachment.

## Diabetic macular oedema

In high-income settings, diagnosis of macular oedema is made by optical coherence tomography (OCT). This uses low power lasers to create a cross-sectional image of the macula, and it is very easy to determine whether or not there is oedema at the fovea. In the absence of OCT, eye workers have to be a bit smarter! If the visual acuity is good (6/12 or better), it is unlikely there is significant macular oedema. If the visual acuity is reduced, then a careful examination of the macula, with a well-dilated pupil and a 60D lens, will usually show swelling, with cysts in the retina. There are also likely to be exudates, small intraretinal haemorrhages, and microaneurysms within 1 disc diameter of the fovea.

The best management of macular oedema is regular injections of an anti-VEGF drug. This treatment has been shown to be more effective than laser in multiple clinical trials. A course of injections improves vision by an average of 10 letters after one year. In comparison, laser treatment may preserve visual acuity, but will not improve it.

## Proliferative diabetic retinopathy

New vessels are usually found on the optic disc or in the mid-peripheral retina, a little beyond the vascular arcades.

They can be detected by carefully examining the retina. Red-free light makes the abnormal vessels easier to detect. The best way of finding these new vessels is with a wide field camera, but these are not always available. Fortunately, new vessels can be treated. If they are at a relatively early stage, with no vitreous haemorrhage or traction retinal detachment, laser treatment will make them shrivel up, which greatly reduces the risk of vision loss. Laser treatment is not directed at the new vessels themselves, but at the peripheral retina. This reduces the production of the growth factors, and the new vessels regress.

### Laser treatment

Laser treatment can be delivered at a slit lamp, or using an indirect ophthalmoscope. Slit lamp laser is quick, but it requires specialist lenses and a delivery system – which is expensive. Laser treatment can also be delivered using an indirect ophthalmoscope, if available, and if the ophthalmologist has the skills to use it. It does not require any additional equipment apart from the laser itself. In this procedure, the patient is lying down and can be given a local anaesthetic block, which makes it easier to do a lot of laser in one session.

Complete laser treatment requires between 2,000 and 3,000 laser burns using a 200-micron laser. Ideally, treatment should be separated into two sessions, about 4–8 weeks apart. This is safer than doing all the laser at once. Too much laser in a short time can lead to rapid fibrosis and contraction of the fibrovascular membranes and may cause a traction detachment.

### Anti-VEGF injections vs laser

Injections of anti-VEGF drugs also cause rapid regression of new vessels. Some trials have shown that the visual results of regular anti-VEGF injections are slightly better than laser treatment. However, laser usually has to be delivered only twice – and it can be done in a single treatment session – but anti-VEGF drugs have to be given every 4–8 weeks, may need to be continued indefinitely, and ideally require an OCT machine to help plan the treatment. Lasers are expensive, and most can only be in one place at a time. They have a finite life expectancy, so even if you have a laser, you may need to replace it after 10 years. Although anti-VEGF drugs are costly, they can potentially be given by a trained nurse, which is already happening in high-income settings, and only requires a clean room. In a low-income setting, the cost of buying a laser is borne by the facility, while the patient has to pay for the cost of the anti-VEGF drugs.

As the cost of anti-VEGF drugs decreases, their role in LMICs is likely to become more prominent. Any clinic that does cataract surgery can give anti-VEGF. In high-income settings, where lasers are widely available, laser remains the treatment of choice for diabetic new vessels, and anti-VEGF is used if there is concurrent macular oedema.

## Vitreoretinal surgery

Despite adequate laser or anti-VEGF treatment, some patients will lose vision because of vitreous haemorrhage or traction detachment of the retina. The best chance of restoring their vision is a vitrectomy. This is complex surgery in which a small (usually 25G) vitreous cutter is used to dissect the vitreous and associated fibrovascular tissue from the retina. This removes any haemorrhage, leaving a clear visual axis, and releases traction on the retina, allowing the retina to re-attach.

**“Early recognition and diagnosis of DR has a major impact on the effectiveness of treatment.”**

The results of vitrectomy for DR have improved greatly over the past two decades. This is partly due to improved technology, as the latest vitrectomy machines are safer. Another important advance is the use of preoperative anti-VEGF injections. This is particularly important in low-resource countries where patients may not have had good laser treatment before surgery. A study in Tanzania showed that pre-treatment with anti-VEGF doubled the probability of restoring sight in eyes blinded by DR. If anti-VEGF is given preoperatively, it should be between 3 and 10 days before surgery. It needs a few days to take effect, but a longer period risks excessive fibrosis and contraction of the membranes, leading to a worse traction detachment.

### Who should be referred?

Because vitrectomy requires complex and costly equipment, only a few centres will be able to offer it. Most eye workers will not need to know the details of the operation, but they do need to know who to refer. The two main indications for surgery are:

- Non-clearing vitreous haemorrhage
- Traction retinal detachment.

Recent haemorrhages (less than one month in duration) can be left to clear, as many will improve with time. If the patient is blind in the other eye, earlier referral and surgery may be indicated. Mild haemorrhages (visual acuity better than 6/36) can also be left, as they will usually clear. Patients with dense haemorrhages, that have not cleared in more than a month, should be considered for vitrectomy.

Traction detachment affecting the macula needs urgent repair, and should be referred immediately. If the detachment is outside the vascular arcades, surgery is not needed . Traction detachments close to the macula, but with good vision, may need surgery. If the patient had laser treatment years ago, the traction is unlikely to get worse, however, if the laser treatment started just a few months ago, the traction detachment is likely to increase, and vitrectomy may be indicated.

## Looking to the future

A decade ago, DR was the leading cause of blindness in people of working age in some high-income countries, like the UK. Thanks to improvements in early detection and management of DR, ophthalmologists in the UK can now assure new patients with retinopathy that – provided they attend the clinic – there is very little risk that they will become blind because of their diabetes.

In low-resource, settings, diabetes is rapidly increasing, and diabetic retinopathy will become an important cause of visual impairment. Although most low- and middle-income countries do not yet have the resources to implement a nationwide screening programme, we need to start putting in place the systems for early detection and treatment now, so that these can be the foundations for even more effective programmes in the future.

